# MNNMs Integrated Control for UAV Autonomous Tracking Randomly Moving Target Based on Learning Method

**DOI:** 10.3390/s21217307

**Published:** 2021-11-02

**Authors:** Mingjun Li, Zhihao Cai, Jiang Zhao, Yibo Wang, Yingxun Wang, Kelin Lu

**Affiliations:** 1 School of Automation Science and Electronic Engineering, Beihang University, Beijing 100191, China; yingy_li@buaa.edu.cn (M.L.); czh@buaa.edu.cn (Z.C.); jzhao@buaa.edu.cn (J.Z.); 2275519001@buaa.edu.cn (Y.W.); 2 Unmanned System Research Institute, Beihang University, Beijing 100191, China; 3 School of Automation, Southeast University, Nanjing 214135, China

**Keywords:** unmanned aerial vehicles, autonomous tracking target, perception and control, integrated controller, multi-neural-network modules, deep learning, reinforcement learning

## Abstract

In this paper, we investigate the problem of unmanned aerial vehicles (UAVs) autonomous tracking moving target with only an airborne camera sensor. We proposed a novel integrated controller framework for this problem based on multi-neural-network modules (MNNMs). In this framework, two neural networks are designed for target perception and guidance control, respectively. The deep learning method and reinforcement learning method are applied to train the integrated controller. The training result demonstrates that the integrated controller can be trained more quickly and efficiently than the end-to-end controller trained by the deep reinforcement learning method. The flight tests with the integrated controller are implemented in simulated and realistic environments, the results show that the integrated controller trained in simulation can easily be transferred to the realistic environment and achieve the UAV tracking randomly moving target, which has a faster motion velocity. The integrated controller based on the MNNMs structure has a better performance on an autonomous tracking target than the control mode that combines with a perception network and a proportional integral derivative controller.

## 1. Introduction

In recent years, the rapid development of unmanned aerial vehicles (UAVs) technology has led to the widespread application of UAVs in a variety of fields and missions, such as natural disaster rescue [1], environment exploration [2], and military reconnaissance [3]. In many application scenarios, the UAVs are required to have the ability to track moving targets [4]. At the same time, as the mission environment for UAVs has become increasingly complex and challenging, the UAV technology will be further developed toward autonomous control with limited payload and information [5].

In the paper, we consider the problem of UAV autonomous tracking moving targets with a single camera sensor. Previous research into UAV tracking targets only considered the guidance controller and assumed that the location of the target was available to the UAV. The conventional feedback control methods or the optimization methods were applied to design the guidance controller. The mainstream method is the proportional-integral-derivative (PID) controllers [6,7]. But the PID controller cannot cope with various tracking situations, and for each case, it requires tedious gain tuning to obtain satisfactory results. Therefore, the PID method does not have a good generalization capability. Other advanced methods such as the sliding model [8], model predictive control [9] and fuzzy PID [10] are also used to design the guidance controller. These methods have higher requirements of the controller input signal and the accuracy of the model.

However, only based on the guidance controller cannot achieve the UAV autonomous tracking target because the UAV cannot autonomous obtain the information of the target. In recent research, computer vision algorithms based on deep learning (DL) [11,12] are used for the UAV to autonomously perceive the target. In this way, the UAV can determine whether the target is within the perception range (the field of view (FOV) of the airborne camera) [13], estimate the partial state of the target [14], and even build a digital map of the surrounding environment [15]. In [16], combining the computer vision method and the traditional control method to achieve the UAV autonomous tracking target.

For the problem described in this paper, the target state that the UAV can observe is limited and poor as there is only one camera sensor to perceive the target. Commonly, the UAV can only estimate the location information of the target, while the velocity and acceleration information of the target is very hard to obtain. Even though it is possible to obtain more data by some complex estimation and filtering algorithms, this data will still contain high noise and uncertainty, which will greatly affect the effectiveness of these control methods.

Therefore, a more robust and adaptive tracking controller is required. Recent research on reinforcement learning (RL) provides a feasible method for this problem. The RL method is an ideal solution for those problems with dynamic systems and uncertain circumstances. Some researches on RL demonstrate that the RL method has been successfully applied to robotics manipulation [16] and motion [17]. Combining RL and DL, some researchers applied the deep reinforcement learning (DRL) method to train the end-to-end controller for the UAV tracking target [18,19]. For the DRL method, only a deep neural network is trained to realize both perception and guidance control. But these are some critical challenges for the DRL method. Training a deep neural network for a complex problem that maps an image directly to the continual control command is very difficult. This learning process requires a large amount of empirical data and spends a lot of time. To increase the effectiveness of training end-to-end controllers, In Ref. [20], the author pre-trains a feasible perception network instead of the part of the end-to-end controller. In Ref. [21], they use the Deep Q-Network (DQN) algorithm and the output of end-to-end control is discrete action. Most importantly, as the end-to-end controller has to train in the simulation environment, the gap between simulation and reality leads to poor performance when the end-to-end controller is transformed to the real world. In most of the related work, their results are only tested in the simulation without the realistic flight tests [22].

In this paper, we propose an integrated neural network framework based on multi-neural-network modules (MNNMs) for a UAV autonomous tracking moving target. This framework divides the neural network controller into two neural networks, which realize the function of target perception and guidance control respectively. The deep learning method is used to train the perception network, and the reinforcement learning method is used to train the guidance control network. The perception network and the tracking control network are perfectly combined into an integrated controller for the UAV autonomous tracking moving target. On the one hand, the training process of the integrated controller will be more efficient and faster than the end-to-end neural network controller training by deep reinforcement learning. On the other hand, the perception network and guidance control network have independent running cycles. The guidance control network can have a faster control frequency without being affected by a long time spent on the perception network, thus improving the accuracy of the UAV tracking target.

In addition, we propose a novel approach to tracking the guidance controller, which does not rely on the accurate dynamic model. In this approach, we transform the more complex and random problem of UAV tracking moving target into the general problem of UAV tracking stationary targets. This approach can further improve the training effectiveness and generalization of the guidance controller., and the resulting network still has a good tracking performance in the case of UAV tracking randomly moving targets.

Finally, the simulation tests in simulation environments and flight tests in realistic environments demonstrate the effectiveness, advantages, and transferred ability of the proposed approach in this paper. In particular, our flight test in the real world allows the UAV to autonomously track the more rapid random motion target, exceeding the demonstrated realistic flight results in most similar articles [16,20,21].

The remainder of this paper is organized as follows. In Section 2, we define the problem of a UAV tracking moving target and describe the basic principles of the deep learning and reinforcement learning methods. In Section 3, we introduce the main framework of the integrated neural network controller and the details of the approach we used to train it. In Section 4, we display the training results and the tracking performance in the simulated environment. In Section 5, we show the tracking performance of flight tests in a realistic environment.

## 2. Preliminaries

### 2.1. Problem Definition

**Figure 1:**
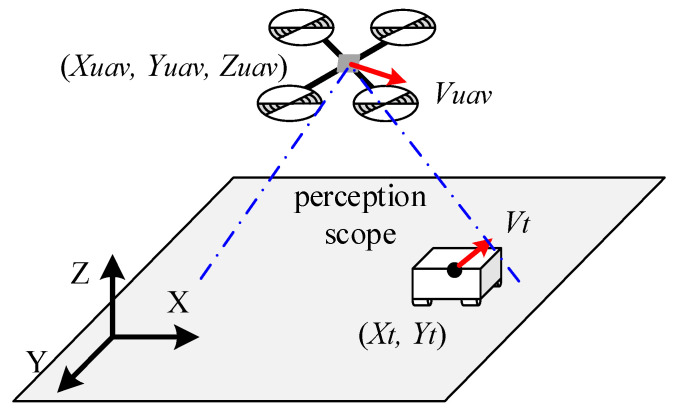
The schematic diagram of the unmanned aerial vehicle (UAV) tracking target problem.

A UAV and target tracking problem is described in this subsection, shown in Figure 1. In this problem, a target is moving on the ground, a UAV autonomously perceives this target through a downward-looking camera mounted underneath the UAV and generates the control commands to track the target. The UAV maintains a certain flight altitude during target tracking. In this problem, the UAV and target are considered as particles relative to the broad tracking environment.

The movement of the UAV can be described by its location and velocity, noted as $S_{uav}=\left[X_{uav},\;Y_{uav},\;Z_{uav},\;V_{uav}\right]$, and the velocity vector is decomposed as $V_{uav}=\left[v_{ux},\;y_{uy},\;z_{uz}\right]$. Since the UAV always keeps at a constant altitude in this tracking system, the vertical velocity of the UAV $z_{uz}=0$. The motion equations of UAV can be expressed as Equation (1).

(1) $$\{\begin{array}{c} {\dot{X}}_{uav}=v_{ux}\\ {\dot{Y}}_{uav}=v_{uy}\\ Z_{uav}=C\;\left(C\;\mathrm{is}\;a\;\mathrm{constant}\right) \end{array}$$

We must take this factor that the variation of velocity is limited by the maximum acceleration that the UAV can achieve. Otherwise, it will result in the situation that the UAV can track the target in the simulation environment but cannot track the target in the real world with the same conditions on target motion. In this work, the UAV is a quadrotor, and it has the same maximum acceleration of the *x*-axis and *y*-axis, noted as $a_{max}$.

(2) $${\dot{v}}_{ux},\;{\dot{v}}_{uy}<a_{max}$$

Similarly, the motion of the target can be described as the location $\left[X_{t},\;Y_{t}\right]$ and the velocity $V_{t}=\left[v_{tx},\;v_{ty}\right]$. However, for the UAV autonomous tracking system, the state of the target is not directly accessible for the UAV. The UAV has to acquire the information of the target by its sensor. We define the relative location of the UAV and the target is $\left[E_{x},\;E_{y}\right]$.

(3) $$\{\begin{array}{c} E_{x}=X_{t}-X_{uav}\\ E_{y}=Y_{t}-Y_{uav} \end{array}$$

### 2.2. Learning Method

With the development of computer vision and machine learning technology, learning-based methods are playing an increasingly significant role in the field of UAV autonomous perception and control [23]. The deep learning method and reinforcement learning method are applied to train the controller for the UAV tracking target. The controller is also known as an agent in the field of machine learning.

#### 2.2.1. Deep Learning

Autonomous perception is the prerequisite for UAV autonomous UAV tracking target. By contrast with the previous research on the UAV tracking target that the UAV directly acquires information about the target by receiving information from outside, the UAV has to determine whether the target is within its field of view and estimate the state of target by its algorithm and sensor. As described in Section 2.1, the airborne camera is the only sensor for UAV perception, and the UAV can obtain RGB images about the environment through the camera.

The convolutional neural network (CNN) has the ability of representation learning. After effective training by the deep learning method, the CNN can transform the abstract image information into straightforward and concrete digital information. In this way, the agent can understand the specific information contained in the image. There are many deep learning algorithms for target detection based on CNN structure, such as Region-CNN (R-CNN) [24], Faster R-CNN [25], and You Only Look Once (Yolo) [26]. Each of them can make the agent detect the specific target in images. The Yolo algorithm has a higher detection frequency among these algorithms, with the sacrifice of detection accuracy. In this work, the Yolo-v3 algorithm [27] is applied to train the perception neural network. This algorithm inherits the advantages of the Yolo algorithm and improves the detection accuracy.

#### 2.2.2. Reinforcement Learning

The UAV tracking target is essentially a sequential decision-making problem. Here, we applied the reinforcement learning method to train a neural network policy for UAV control decisions. RL methods are divided into two classes: value-based and policy-based. Since the control of UAV is a continuous control problem, the policy-based proximal policy optimization (PPO) [28] is applied in this paper. the PPO algorithm is known to outperform other state-of-the-art RL algorithms in challenging problems, and the PPO algorithm is the default algorithm for Open-AI when solving complex problems.

At each control cycle, the agent of UAV receives a state $s_{t}$ from the airborne sensor and generates a control command (action) $a_{t}$ according to the policy π. Once the agent executes the control command $a_{t}$, the state will be changed and in return, produce a reward $r_{t}=R\left(s_{t},\;a_{t},\;s_{t+1}\right)$ to indicate the performance of this action. In one episode, a sequence of interactions in the time series can be represented by a trajectory: $\tau =s_{0},\;a_{0},\;r_{0},\;\ldots ,\;s_{t-1},\;a_{t-1},\;r_{t-1},\;s_{t},\;\ldots$. The agent will obtain a cumulative reward: $R_{t}=\underset{t}{\sum }r_{t}$. Based on the cumulative reward $R_{t}$, there are two important value functions to evaluate a policy π: the V-function $V_{\pi }\left(s_{t}\right)$ and the Q-function $Q_{\pi }\left(s_{t},a_{t}\right)$.

(4) $$V_{\pi }\left(s_{t}\right)=E{\left[R_{t}|s_{t}=s\right]}_{\pi }$$

(5) $$Q_{\pi }\left(s_{t},a_{t}\right)=E{\left[R_{t}|s_{t}=s,a_{t}=a\right]}_{\pi }$$

$V_{\pi }\left(s_{t}\right)$ represents the expected value of $R_{t}$ when the agent is in state $s_{t}$ and under policy π. $Q_{\pi }\left(s_{t},a_{t}\right)$ represents the expected value of $R_{t}$ when the agent take action $a_{t}$ in state $s_{t}$ and under the policy π. The goal of the RL method is to train the policy π to maximize the expected value of the cumulative reward.

## 3. Integrated Controller Training by Learning Method

### 3.1. Controller Framework Based on Multi-Neural-Network Modules (MNNMs)

UAV autonomous tracking dynamic target only based on raw image information is a complex and challenging problem for deep reinforcement learning. It is not easy to directly train a whole neural network mapping a relationship from image to action control of the UAV. In this work, we design a feasible approach for this problem. We propose an integrated neural network control framework based on multi-neural-network modules (MNNMs). Unlike the end-to-end neural network, the integrated neural network controller consists of several neural network modules, and each module is designed for a different function.

**Figure 2:**
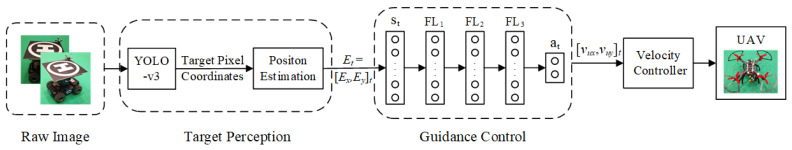
The integrated controller framework for UAV tracking target based on multi-neural-network modules (MNNMs).

In this work, we divide the problem of the UAV autonomous tracking target into two main functions, which are target perception and guidance control. Then, two neural network modules are designed for the two functions, shown in Figure 2. In the perception part, the UAV autonomously processes the image acquired from the airborne camera and estimates the relative distance between the UAV and target. The results of target perception are the input of the guidance control part, and its output is the velocity command of the UAV. For the integrated neural network controller, it can map a relationship between the raw image information to the continuous control commands of the UAV. This tracking target controller can be applied to various UAVs as long as they have an inner-loop velocity controller that responds to the velocity command.

### 3.2. Target Perception

**Figure 3:**
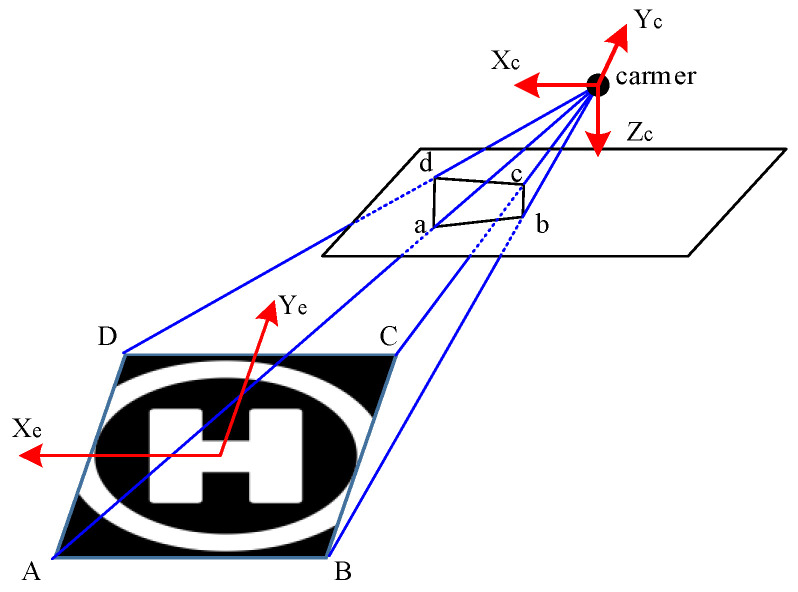
Concept diagram of perspective-n-point (PNP) problem.

In the target perception part, the input is the raw image from the airborne camera and the output is the estimate of the relative distance between the UAV and the target at the time of this image. This process consists of target detection and position estimation. We apply the Yolo-v3 algorithm to train a deep neural network for a specific target perception, as long as we provide an extensive enough dataset for this target. Through the target perception, the UAV can determine whether the target is within the FOV of the airborne camera. If the target is within the image, the perception network will also output four vertices’ coordinates of a rectangle in the image pixel coordinate system. This rectangle exactly frames the target and represents the position and size that the target occupies in this image. Then, the UAV can estimate the relative position between the UAV and the target based on these data. There is a perspective-n-point (PNP) problem [29], shown in Figure 3.

Consider a point “A” in the world coordinate system, and point “a” is the corresponding point of “A” in the camera image. We note that the coordinate of “A” in the world coordinate system is $T_{w}$, and the coordinate of “a” in the camera coordinate system is $T_{c}$, then $T_{c}$ has the following mapping relationship with $T_{w}$:

(6) $$T_{c}=R_{cw}\times T_{w}+t_{cw}^{c}$$

where $R_{cw}$ is the rotation matrix from the world coordinate system to the camera coordinate system, and $t_{cw}^{c}$ is the translation vector from the origin of the camera coordinate system to the origin of the world system in the camera coordinate system. As shown in Figure 3, We consider the center of the tracking target to be the origin of the world coordinate system and we can get the four vertexes coordinates $\left[\begin{array}{cc} \begin{array}{cc} A & B \end{array} & \begin{array}{cc} C & D \end{array} \end{array}\right]$ of the target in the world coordinate system. Through the perception network, we can get the corresponding coordinates of points $\left[\begin{array}{cc} \begin{array}{cc} a & b \end{array} & \begin{array}{cc} c & d \end{array} \end{array}\right]$ in the pixel coordinate system. The coordinates of $\left[\begin{array}{cc} \begin{array}{cc} a & b \end{array} & \begin{array}{cc} c & d \end{array} \end{array}\right]$ in the pixel coordinate system can be converted to the coordinates in the camera coordinate system according to the internal parameters of the camera. Then the P3P algorithm [30] is applied to solve for $t_{cw}^{c}$. The relative distance vector in the world coordinate system between the UAV and the target can be calculated as Equation (7).

(7) $$t_{bw}^{w}=R_{wb}\times \left(R_{bc}\times t_{cw}^{c}\right)+t_{bc}^{w}$$

where, $R_{bc}$ is the rotation matrix from the camera coordinate system to the body system of the UAV, and the $R_{wb}$ is the rotation matrix from the body coordinate system to the world coordinate system. $t_{bc}^{w}$ is the translation vector from the center of the UAV to the camera in the world coordinate system. $R_{bc}$ and $t_{bc}^{w}$ is related to the location of camera mounting on the UAV, and $R_{wb}$ is related to the attitude of the UAV.

Typically, the resultant area of the target detection in the image is slightly larger than the actual area occupied by the target. This error will lead to the inaccuracy of relative distance estimation between the target and the UAV indirectly. Therefore, higher performance is required for the guidance controller.

### 3.3. Guidance Control

In this subsection, the reinforcement learning method is used to train the guidance controller for the UAV autonomous tracking target. The guidance controller is essentially a multilayer neural network shown in Figure 2. The input of the guidance controller is the output of the perception part. As the UAV only estimates the relative distance between the UAV and the target, the input of the guidance controller is the relative distance for the five latest moments.

$$s_{t}=\left[\begin{array}{cc} \begin{array}{cc} E_{t-4} & E_{t-3} \end{array} & \begin{array}{ccc} E_{t-2} & E_{t-1} & E_{t} \end{array} \end{array}\right]$$

where $E_{t}={\left[\begin{array}{cc} E_{x} & E_{y} \end{array}\right]}_{t}$, denotes the relative distance between UAV and target along the *x*-axis and *y*-axis at moment t. The output of the guidance controller is the expected velocity along the *x*-axis and *y*-axis of the UAV, $a_{t}={\left[\begin{array}{cc} u_{vx} & u_{vy} \end{array}\right]}_{t}$.

**Figure 4:**
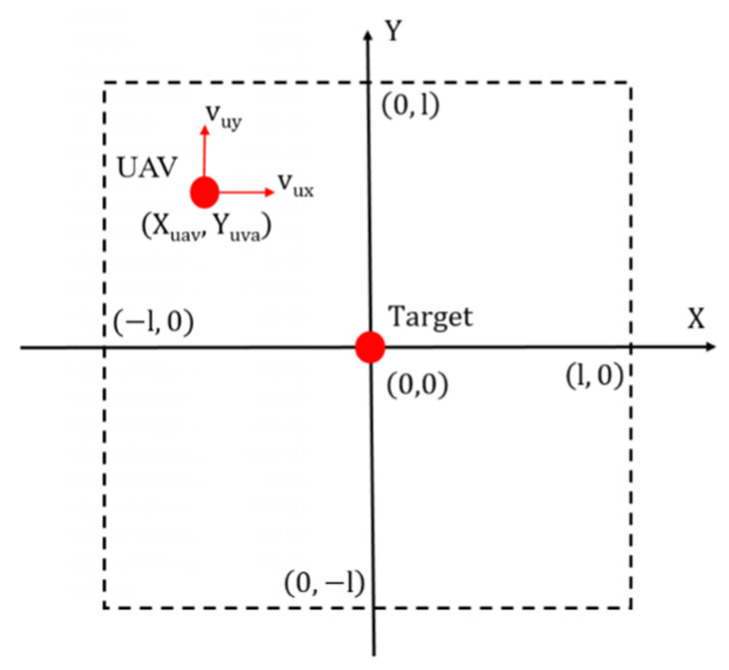
Training model for UAV target tracking problem.

Since the training process of the reinforcement learning method has to be realized in simulation, the simulation model of the interaction between the agent and environment has to be built. However, it is challenging to build a dynamic model for UAV tracking the randomly moving target. Here, we introduce a novel training idea to train the guidance controller that maps the relationship between the relative distance and velocity command. For the UAV tracking target problem introduced in Section 2.1, the control command of the guidance control is essentially generated by the relative distance. The random trajectory of the target can be regarded as a series of points. Suppose the guidance controller can generate the correct action $a_{t}$ at time *t*, to allow the UAV to track the target’s current. In that case, the UAV can track the target in the whole time series under the control of the tracking controller. Based on this idea, we can turn the problem of UAV tracking randomly moving the target into the problem of the UAV tracking a stationary target. According to this idea, an effective training model is shown in Figure 4.

In this training model, the motion of the UAV and target are limited in a 2l×2l area. For any training episode, the target stays at the center of this area. The UAV is located anywhere within the area allowed at the initial moment of an episode. The objective of the tracking controller is to make the UAV fly toward the target from any location as soon as possible. According to the objection of the guidance controller, the reward function of this problem is given as Equation (8).

(8) $$\begin{array}{c} r_{t}=-\left({||E_{t+1}||}_{2}+0.1*{||a_{t}||}_{2}\right)\\ r_{t}=-\left({||E_{t+1}||}_{2}+0.1*{||a_{t}||}_{2}\right)-50\;if\;\left(\left|X_{uax}\right|>l\;or\;\left|Y_{uax}\right|>l\right) \end{array}$$

The reward function is mainly related to the relative distance between the UAV and the target. Action $a_{t}$ will acquire a higher reward if it shortens the relative distance. A minor penalty is imposed on the action to prevent the policy to make more aggressive commands. If the UAV is out of the limited area, the action will receive a significant penalty additionally. The value of the penalty is 50.

**Figure 5:**
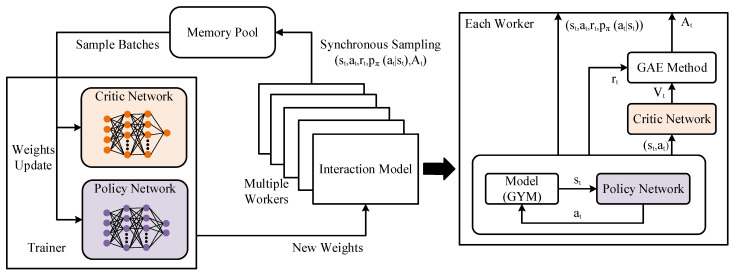
The training process of guidance controller by the distributed reinforcement learning (RL) Batch-PPO (proximal policy optimization) algorithm.

A digital model for the tracking process described above is built based on the GYM library [31] to train the guidance controller. The model is completed in python language. The Rllib [32] library is applied to realize the training process. The Rllib is an open-source library to realize the RL methods and supports distributed training. It offers a unified API to choose different RL algorithms and tune hyperparameters. For distributed RL training, there are multiple parallel workers. The interaction between the environment and the agent runs simultaneously in each worker. In the Batch-PPO algorithm, there are two neural networks. One is the policy network for the agent to make a decision, another is the critic network to estimate the V-function. The policy network and critic network for each worker are the same and will be changed after each weights update. The training process of the tracking controller is shown in Figure 5.

Consider a continuous trajectory of one episode experience data in one worker: $s_{0},\;a_{0},\;r_{0},\;s_{1},\;a_{1},\;\ldots ,\;s_{t},\;a_{t},\;r_{t},\;\ldots ,\;a_{T-1},\;r_{T-1},\;s_{T}$, *T* is the length of the trajectory. One piece of experience data for a moment *t* can be written as $\left[s_{t}\;\;\;a_{t}\;\;\;r_{t}\;\;\;p_{\pi }\left(a_{t}|s_{t}\right)\;\;\;A_{t}^{\pi }(s_{t},a_{t})\;R_{t}\;V\left(s_{t}\right)\right]$. Where, $p_{\pi }\left(a_{t}|s_{t}\right)$ represents the probability of the agent choosing the action $a_{t}$ according to the current state $s_{t}$ and the current policy π. $V\left(s_{t}\right)$ is the V-function that is estimated by the critic network. $R_{t}$ is the cumulative reward $R_{t}=\overset{T}{\underset{i=t}{\sum }}\gamma^{i-t}r_{i}$, where γ is the discount factor. $A_{t}^{\pi }\left(s_{t},a_{t}\right)$ is the advantage function (A-function), represents the relative advantage value of the agent choosing the action $a_{t}$ better than a general action under the current state and policy. A-function is defined as: $A_{t}\left(s_{t},a_{t}\right)=Q\left(s_{t},a_{t}\right)-V\left(s_{t}\right)$. The generalized advantage estimator method [33] is used to estimate the A-function as Equations (9) and (10),

(9) $$A_{t}\left(s_{t},a_{t}\right)=\delta_{t}+\left(\gamma \lambda \right)\delta_{t+1}+\ldots +{\left(\gamma \lambda \right)}^{T-t+1}\delta_{T-1}$$

(10) $$\delta_{i}=r_{i}+\gamma V\left(s_{i+1}\right)-V\left(s_{i}\right),\;i=0,\;1,\;\ldots ,\;T-1$$

where λ is the compromise coefficient between the bias and variance of the A-function. The experience data generated from all workers is sent into the memory pool. When the memory pool is full, the policy network and the critic network will be updated according to the Batch-PPO algorithm. The batch size is *K*. At each update time, *K* piece of data is randomly selected from the memory pool. the policy network is updated by the performing gradient ascent based on Equations (11) and (12).

(11) $$\theta_{k+1}=argmax_{\theta }\frac{1}{num\left(\tau \right)}\sum_{\tau }\left[\frac{1}{K}\overset{K}{\underset{t=0}{\sum }}L\left(s_{t},\;a_{t},\;\theta_{k},\;\theta \right)\right]$$

(12) $$L\left(s_{t},\;a_{t},\;\theta_{k},\;\theta \right)=min\left[\begin{array}{cc} \frac{\pi_{\theta }\left(a_{t}|s_{t}\right)}{\pi_{\theta_{k}}\left(a_{t}|s_{t}\right)}A^{\pi_{\theta_{k}}}\left(a_{t}|s_{t}\right) & clip\left[\begin{array}{ccc} \frac{\pi_{\theta }\left(a_{t}|s_{t}\right)}{\pi_{\theta_{k}}\left(a_{t}|s_{t}\right)} & 1-\varepsilon & 1+\varepsilon \end{array}\right]A^{\pi_{\theta_{k}}}\left(a_{t}|s_{t}\right) \end{array}\right]$$

where θ is the weights of the policy network, and $\theta_{k}$ represents the current weights and $\theta_{k+1}$ represents the next time weights after an update. ε is the clip value. the critic network is updated as follows to minimize the mean square error between the cumulative reward and the estimated value of the V-function, where ω is the weight of the critic network.

(13) $$\omega_{k+1}=argmin_{\omega }\sum_{K}{\left[R_{i}-V_{\omega }\left(s_{i}\right)\right]}^{2}$$

After one iteration of the weights update is over, the memory pool is cleared. The workers use the updated policy network and critic network to generate new experience data.

## 4. Experiments and Simulation Results

In this section, we implement our proposed approach in the simulation environment, including the training process and results of the integrated controller, the tests of the integrated controller trained by our approach. The operating system utilized for running the integrated controller training and simulated tests is Ubuntu 16.04, with the GPU: Nvidia GeForce RTX 2080 and CPU: Intel twelve-core i7-8750H.

### 4.1. Training Results

#### 4.1.1. Perception Neural Network

**Figure 6:**
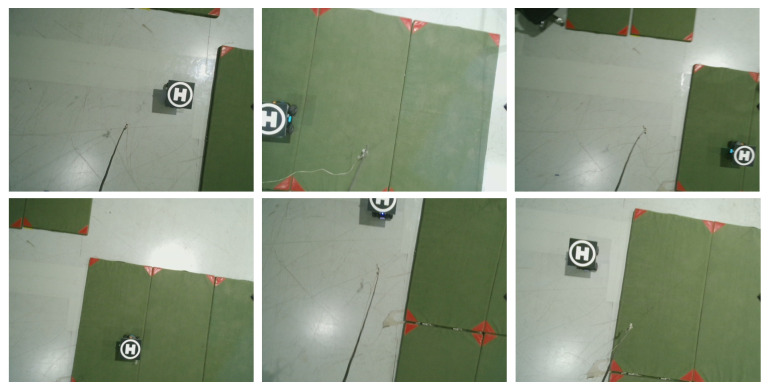
Part of the dataset for perception network training.

An “H-shaped” marker is selected as the identifier of the target. To train an effective deep neural network to perceive this “H-shaped” marker, we specifically make a calibrated dataset for it in real-world scenarios. To make the perception neural network adapt to more application scenarios, the dataset is collected with different flight altitudes, and some images of the dataset only contain a partial target. There are about 400 images in this dataset, some of them shown in Figure 6. We use the default network structure and hyperparameters of the Yolo-v3 algorithm to complete the training of the perception neural network. During the training process, 80% of the dataset is used for training, and 20% of the dataset is used for testing.

The training process executes 10,000 iterations and takes about 5 h. Finally, the average loss of the test dataset is stably less than 0.05. The 0.05 is an experience value for the Yolo-v3 algorithm. When the average loss is less than 0.05, the result deep neural network has a good perception ability for the specified target. The result deep neural network is used as the perception neural network for the integrated tracking controller. As the dataset for training is sampled in a realistic environment, the result of the deep neural network has a good target detection ability for the “H-shaped” marker both in the simulated and realistic environments.

#### 4.1.2. Guidance Control Neural Network

In this subsection, the Batch-PPO algorithm is applied to train the policy for guidance control individually. The policy for guidance control is a neural network. The resulting neural network will be used as the guidance controller, which is a part of the integrated controller. As described in Section 3.3, the random tracking problem is simplified to a normalized tracking problem. The number of the state and action of this problem is not too large, and the value range of this problem is limited. In this way, the structure of the policy for this problem is not required to be too complex, and a fully connected neural network with three hidden layers is enough. The nodes of the hidden layers are $\left[\begin{array}{ccc} 128 & 128 & 64 \end{array}\right]$. The activation function of the hidden layers is the relu function and the activation function of the output layer is the tanh function. Actually, we use kinds of policies with different numbers of hidden layers and nodes to train. The experimental results show that this structure is sufficient to cope with this problem, the policy with more layers and nodes cannot increase the cumulative reward and create a burden for the training process.

**Figure 7:**
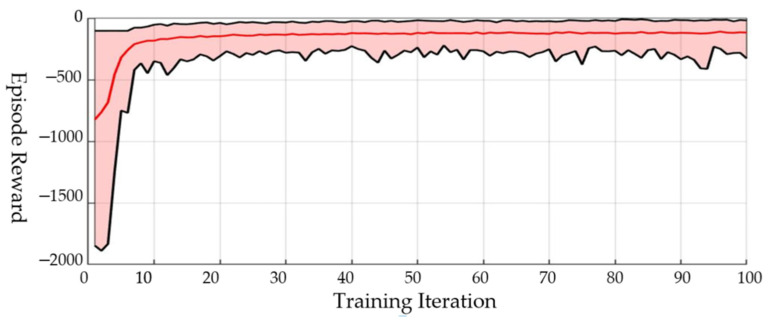
Training result of the guidance control neural network.

The hyperparameters of the Batch-PPO algorithm are also chosen from the experience and series of experiments. Some of the hyperparameters can apply the experience values of the algorithm. However, most of the hyperparameters are determined by a series of training experiments. We use different sets of hyperparameters to train the policy, and choose the value of hyperparameters which can make the agent obtain the maximum cumulative reward when the training process is converged. The hyperparameters we used for the Batch-PPO algorithm are shown in Table 1. The training result is shown in Figure 7. The training process executes 100 iterations and takes about 30 min. However, the training result has largely converged after 10 iterations, and this process takes about 5 min.

### 4.2. Simulation Test

In this section, we display the flight test for a UAV autonomous tracking randomly moving target with the integrated controller trained in Section 4.1 to test the performance of this controller. The test is performed in a simulation environment. The interaction model between the agent and the environment is built based on Gazebo, including a UAV model with an inner-loop controller and a target model. In this environment, the UAV and the target can move freely.

**Figure 8:**
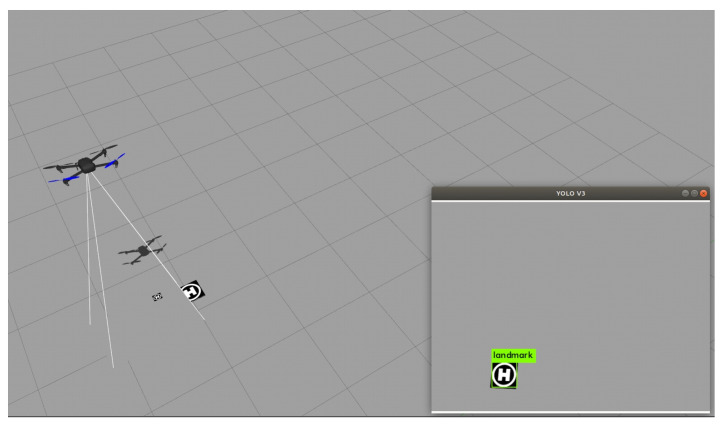
Flight test in the simulation environment.

**Figure 9:**
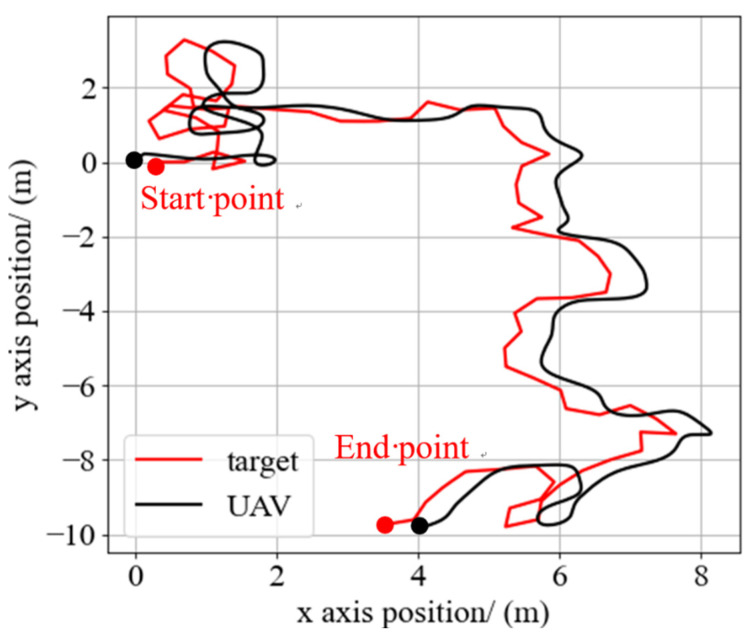
The trajectory of the UAV and target during the tracking process in the simulation test.

At the beginning of the tracking process, the target remains stationary and the UAV hovers above the target autonomously under the control of the integrated controller. The hover altitude of the UAV is 3 m. The target starts to move at any moment, and the UAV tracks the target to ensure it is always within the FOV of the airborne camera. When the target escapes the FOV of the airborne camera, the UAV stop moving and the tracking process fails. During the tracking process, the velocity and motion direction of the target will change randomly every 0.5 s, the velocity of the target is between 0.5~1 m/s. Figure 8 shows the flight test in the simulation environment, and Figure 9 shows the trajectory of the UAV and target in one flight test.

The whole training of the integrated controller takes about 6 h, much less than the end-to-end neural network controller training by the DRL method. At the same time, the flight test in the simulation environment demonstrates that the integrated controller trained by our approach has a good tracking performance on UAV tracking the randomly moving target.

## 5. Flight Results

We train and test the integrated controller for the UAV autonomous tracking target in the simulation environment. In addition to this, we also test the result controller in the real world. Among the many research works on RL, it is very challenging to realize sim-to-real as the gap between the simulation training environment and application environment in the realistic world. However, the integrated controller trained by our approach can be well transferred to the realistic world. Multiple flight tests and comparisons also demonstrate the good tracking performance and robustness of our result controller.

### 5.1. Hardware Platform

**Figure 10:**
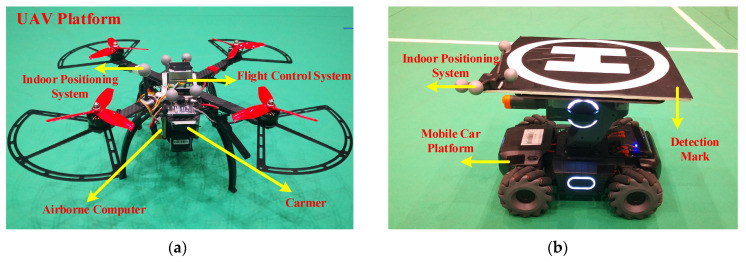
(**a**) The UAV platform is used for flight tests in the real world. (**b**) The target platform is used for flight tests in the real world.

A self-assembled quadrotor, shown in Figure 10a is selected as the UAV platform. The UAV is equipped with a camera, an airborne computer and a flight control computer. The target platform, shown in Figure 10b consists of the DJI Robomaster S1 and an “H-shaped” tracking mark fixed on the top of the car. We select this robot because it can move along the regular trajectory (like straight, square or circle line) and can be controlled to move randomly by a remote controller. The UAV and target are also equipped with the indoor positioning balls, respectively, which cooperate with the optitrack system to record the real-time location of UAV and target during the flight test. The location information of UAV and target will not be used in the tracking process.

**Figure 11:**
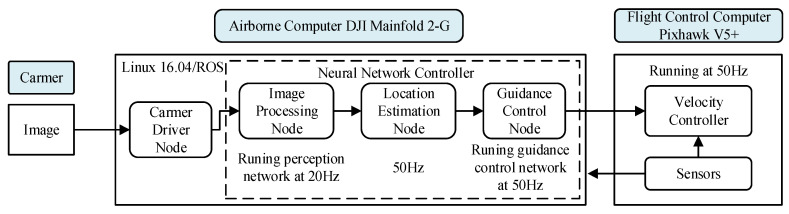
The structure and function of the UAV platform in the flight test.

The structure and functional distribution of the UAV are shown in Figure 11. The airborne computer is a DJI Manifold 2-G with the GPU NVIDIA Jetson TX2, and the integrated controller runs under a robot operating system (ROS). The airborne camera is a common optical sensor with a resolution of 640×480 pixels, 2.8 mm focal length, 62° horizontal FOV and 46° vertical FOV. The deep neural network for target perception runs at a lower frequency of 20 Hz. The location estimation node and guidance control network run at a faster frequency of 50 Hz which is equal to the running frequency of the UAV inner-loop controller. The flight control computer is a Pixhawk V5+, and a velocity controller based on the PID method is run in it to make the UAV respond to velocity command from the airborne computer. The rules of tracking tests are the same as the simulation tests described in Section 4.2, and the flight altitude is also 3 m.

### 5.2. Comparing with Proportional-Integral-Derivative (PID) Method

First, we tested the tracking performance of the integrated controller we trained. Since the integrated controller mainly consists of two neural networks, we also called it the neural network (NN) controller. We compared the NN controller with the common approach that combines perception and conventional feedback control. A PID controller for guidance control was designed instead of the neural network guidance controller we trained, and the perception network and location estimation were the same in both approaches.

**Figure 12:**
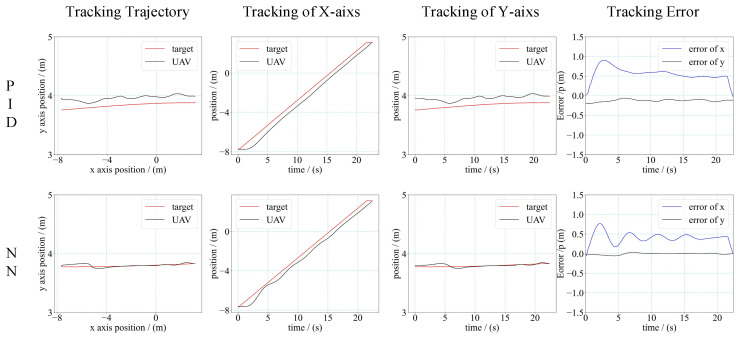
Trajectory and tracking error of UAV tracking target. (Target’s velocity is 0.5 m/s).

**Figure 13:**
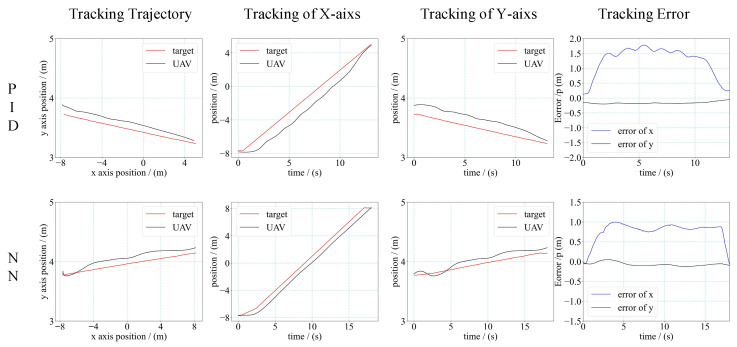
Trajectory and tracking error of UAV tracking target. (target’s velocity is 1.0 m/s).

**Figure 14:**
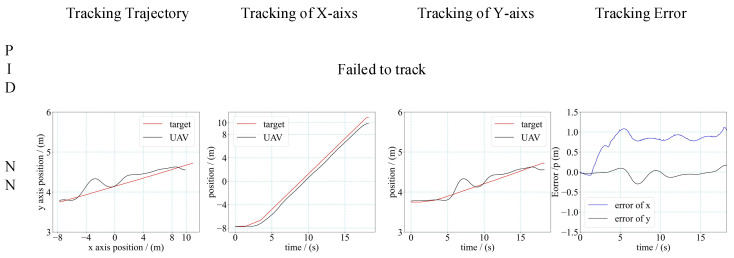
Trajectory and tracking error of UAV tracking target. (Target’s velocity is 1.2 m/s).

We applied a simple mission scenario of the target moving along a straight line. The velocity of the target was gradually increasing from 0.5 m/s to 1.2 m/s in multiple tests. The results of these tests are shown in Figure 12, Figure 13 and Figure 14, including the tracking trajectory (in two-dimensional planar), the tracking performance of *X*-axis and *Y*-axis, the tracking error of *X*-axis and *Y*-axis for the different velocity.

The performance of the controller was evaluated by the mean tracking error (MTE) of the *X*-axis and *Y*-axis, and the steady state error (SSE) during the tracking tests. The mean tracking error was calculated as Equation (14).

(14) $$\mathrm{MTE}=\left(\overset{T}{\underset{0}{\int }}\left|e_{t}\right|dt\right)/T$$

*T* denotes the total time duration of the tracking process, and $e_{t}$ denotes the tracking error of the *x*-axis or *y*-axis at moment *t*. The calculation results are shown in Table 2. From the results, we can see that when the velocity of the target was 0.5 m/s and 1 m/s, the MTE of the NN controller was smaller than the MTE of PID. The SSE was closed for the NN controller and PID controller when the velocity of the target was small. When the velocity increased, the NN controller had a smaller SSE than the PID controller. In addition, the UAV with an NN controller could track the faster moving target than with the PID controller. For the PID controller, the maximum velocity of the target that the UAV could track was 1 m/s. For the NN controller, the maximum velocity of the target that the UAV could track was 1.2 m/s. The flight results demonstrated that the NN controller had higher control accuracy than the PID controller, and had a faster response time to make the UAV track a faster moving target.

It should be additionally noted that the UAV was hovering above the target before it moved. As the target starts to move, the UAV will receive the velocity command from the tracking controller. However, there is a delay for the UAV to fully respond to the velocity command from start to move, which is related to the inherent characteristics of the UAV. Due to this limitation, the UAV found it hard to track a target with faster movement, as the target escaped from the perception range of the UAV before the UAV starts to move.

In addition, the PID controller for the UAV tracking target consumed a lot of time and effort for tuning the parameters during the tests. We had to retune the parameters as the velocity of the target changed. The final PID parameters used in different tests are shown in Table 3. However, the NN controller did not require any tuning for different tests. The results of these flight tests demonstrate that, compared to the PID controller, the NN controller trained by the proposed approach had a better performance on the UAV tracking target, and has better robustness.

### 5.3. Autonomous Tracking Target under Complex Scenario

**Figure 15:**
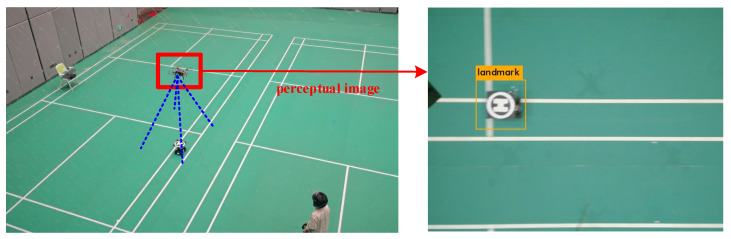
The real-world environment of the flight test.

The flight tests in Section 5.2 showed that the neural network controller trained by our approach enabled the UAV to achieve an autonomous tracking target with the target moving along a straight line. In addition to this, we enabled the NN controller to achieve more complex tracking scenarios, including tracking the target moving along the circle line and moving randomly. Figure 15 shows the real-world environment of the flight test when the UAV tracks the target and the FOV of the airborne camera with the target detection result.

**Figure 16:**
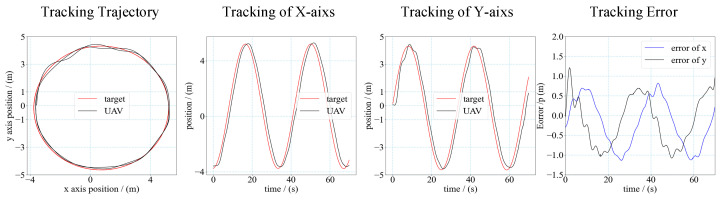
Trajectory and error of UAV tracking target. (The target moves along circle line at a velocity of 1 m/s).

By contrast with the target moving along a straight line, when the target moves along the circle line, it will lead to the velocity command of both the *X*-axis and *Y*-axis. Considering the inertial motion of the UAV, it is more difficult to track a target with circular motion than a target with linear motion. The trajectory and tracking error of the UAV and target of the tracking test is shown in Figure 16. The velocity of the target is 1 m/s. 

**Figure 17:**
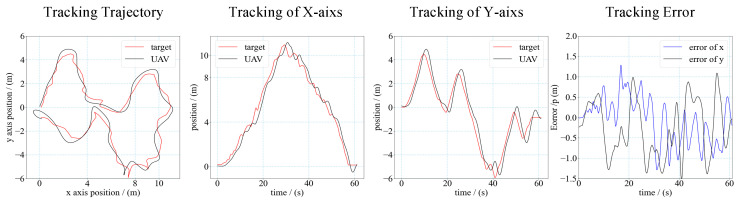
Trajectory and error of UAV tracking target. (The target moves randomly).

In most practical application scenarios, the motion of the target is uncertain and irregular. Therefore, we also tested the tracking performance when the target was moving randomly. In this test, the motion of the target was manipulated by a human through remote control, and the trajectory of the target was completely random. The tracking result is shown in Figure 17. The max velocity of the target reaches 1.2 m/s. The results show that the neural network controller can make the UAV autonomously track the randomly moving target.

As the neural network controller is trained in the simulation environment, the multiple flight tests proved that our neural network controller can be successfully transferred to a realistic environment from the simulation environment. At the same time, the neural network controller has good general performance and can be applied to various tracking scenarios.

## 6. Discussion and Conclusions

In this paper, we propose a feasible approach that applies the machine learning methods for the UAV autonomous tracking target problem. By contrast with the DRL method that directly trains an end-to-end neural network for this problem, we provide a new integrated controller with the MNNM structure. This integrated controller consists of the perception part and the guidance control part. The deep learning method and reinforcement learning method are applied to train the perception network and guidance control network, respectively. Meanwhile, when using the reinforcement learning method to train the guidance controller, we propose a novel approach to achieve UAV tracking of a randomly moving target by only training the guidance controller for UAV tracking stationary target. Through the two approaches, the integrated controller is trained rapidly and efficiently. The training process takes about 6 h, which is much less than the time consumed by the DRL method. In addition, the subsequent flight tests also show that the integrated controller trained by our approach in the simulated environment can be directly transferred to the real environment without any tuning.

To test the tracking performance of this integrated controller, we conducted a series of flight tests. First, we compared the integrated controller with the controller whose guidance control is based on the conventional PID method. We designed a basic tracking task in which the target moved along a straight line with a certain velocity. The results of multiple tests show that the integrated controller has less tracking error and steady state error than the PID method when the velocity of the target is 0.5 m/s and 1 m/s. Moreover, the UAV can track a faster-moving target integrated under the same experimental conditions.

In addition to the comparative test, we tested the integrated controller in more complicated tasks. In our flight tests, the UAV could autonomously track a randomly moving target whose velocity is up to 1.2 m/s. Compared with the videos in similar articles [20,21] which use the DRL method to achieve the UAV autonomous tracking target, the target has a faster velocity and wilder motion range in our flight tests.

A series of flight tests in the simulated and realistic environment demonstrated that the integrated controller trained by our approach can be applied to various tracking scenarios. The moving trajectory of the target is unrestricted, and the velocity of the target is variable within a certain range. For our approach, the maximum velocity of the tracking target is related to the FOV of the airborne camera and the inherent characteristics of the UAV. In our flight tests, when the flight altitude of the UAV was 3 m, the maximum velocity of the tracking target could be 1.2 m/s.

The integrated controller introduced in this paper can be applied to most UAV tracking target tasks as the model described in Section 2.1. However, our approach can only make the UAV track one specified target at one moment in a free-flying environment. The tracking target can be any other target as long as we can provide a sufficient dataset of the target. To achieve a better tracking performance, we suggest that the dataset includes most of the possible states that the target could be in the FOV of the airborne camera.

In the future, we want to improve the ability of the integrated controller to improve the intelligence of the UAV. One limitation of the integrated controller we proposed is that the velocity command is generated without the overall environmental analysis. Therefore, we expect to adjust the training mechanism and the structure of the integrated controller, or add new neural network modules to make the controller adapt to more complicated mission scenarios, such as obstacles or confusing targets in the environments that affect the flight and decisions of the UAV.

## Figures and Tables

**Table 1:** List of hyperparameters for Batch-PPO algorithm.

Hyperparameters	Value	Hyperparameters	Value
no. of workers	10	lamda (*λ*)	0.97
memory size (*N*)	50,000	clip (*ε*)	0.2
batch size (*K*)	2000	actor net learning rate	0.0003
length of one episode (*T*)	500	critic net learning rate	0.0015
gamma (*γ*)	0.998	grad_norm	0.5

**Table 2:** Tracking error of UAV for tracking a line-moving target with different velocity.

Velocity	0.5 m/s	Velocity	0.5 m/s
**Proportional Integral Derivative (PID) Controller**			
**MTE of X (m)**	0.55	1.14	Failed
**MTE of Y (m)**	0.12	0.16	
**SSE (m)**	0.52	1.53	
**Neural Network Controller**			
**MTE of X (m)**	0.46	0.80	0.83
**MTE of Y (m)**	0.02	0.07	0.08
**SSE (m)**	0.41	0.85	0.95

**Table 3:** The PID parameters of flight tests.

	0.5 m/s	1 m/s	1.2 m/s
**P**	1.5	2.2	2.4
**I**	0.04	0.1	0.12
**D**	0.01	0.01	0.01

## Data Availability

Not applicable.
